# Divergent regulation of Arabidopsis *SAUR* genes: a focus on the *SAUR10*-clade

**DOI:** 10.1186/s12870-017-1210-4

**Published:** 2017-12-19

**Authors:** Hilda van Mourik, Aalt D. J. van Dijk, Niek Stortenbeker, Gerco C. Angenent, Marian Bemer

**Affiliations:** 1 Laboratory of Molecular Biology, Wageningen University & Research, Droevendaalsesteeg 1, 6708 PB Wageningen, the Netherlands; 2 Bioinformatics group, Biometris, and Business Unit Bioscience, Wageningen University & Research, Droevendaalsesteeg 1, 6708 PB Wageningen, the Netherlands; 3 Microbial Physiology Group, MPI for Marine Microbiology, Celsiusstr. 1, D-28359 Bremen, Germany; 4 Laboratory of Molecular Biology and Business Unit Bioscience, Wageningen University & Research, 6708 PB Wageningen, the Netherlands

**Keywords:** SAUR, Hormones, Growth, Cell elongation, Regulatory region, Auxin, Brassinosteroids, ABA, Shade response

## Abstract

**Background:**

*Small Auxin-Upregulated RNA* (*SAUR*) genes encode growth regulators that induce cell elongation. Arabidopsis contains more than 70 *SAUR* genes, of which the growth-promoting function has been unveiled in seedlings, while their role in other tissues remained largely unknown. Here, we focus on the regulatory regions of Arabidopsis *SAUR* genes, to predict the processes in which they play a role, and understand the dynamics of plant growth.

**Results:**

In this study, we characterized in detail the entire *SAUR10*-clade: *SAUR8*, *SAUR9*, *SAUR10*, *SAUR12, SAUR16*, *SAUR50*, *SAUR51* and *SAUR54*. Overexpression analysis revealed that the different proteins fulfil similar functions, while the *SAUR* expression patterns were highly diverse, showing expression throughout plant development in a variety of tissues. In addition, the response to application of different hormones largely varied between the different genes. These tissue-specific and hormone-specific responses could be linked to transcription factor binding sites using in silico analyses. These analyses also supported the existence of two groups of *SAURs* in Arabidopsis: Class I genes can be induced by combinatorial action of ARF-BZR-PIF transcription factors, while Class II genes are not regulated by auxin.

**Conclusions:**

*SAUR10-*clade genes generally induce cell-elongation, but exhibit diverse expression patterns and responses to hormones. Our experimental and in silico analyses suggest that transcription factors involved in plant development determine the tissue specific expression of the different *SAUR* genes, whereas the amplitude of this expression can often be controlled by hormone response transcription factors. This allows the plant to fine tune growth in a variety of tissues in response to internal and external signals.

**Electronic supplementary material:**

The online version of this article (10.1186/s12870-017-1210-4) contains supplementary material, which is available to authorized users.

## Background

Plant growth is highly flexible and can be adjusted in response to developmental and environmental cues. Factors controlling these cues, such as transcription factors (TFs), light response factors and hormones, can activate or repress growth regulators that integrate the different signals into a growth response. An important group of growth regulators is formed by the Small Auxin-Upregulated RNAs (SAURs), which were first discovered as small transcripts that were rapidly upregulated in response to auxin [1]. In the absence of growth-inducing signals, SAUR activity can be reduced fast, because transcript and protein half-lives were found to be very short [2]. Over-expression studies using various Arabidopsis *SAUR*s revealed their general capacity to induce cell elongation and growth, whereas knock-out studies yielded little results, probably due to a high level of redundancy amongst the 79 Arabidopsis *SAUR* genes [3–9]. SAURs can induce cell elongation by interacting with PP2C-D phosphatases, thereby inhibiting their phosphatase activity and preventing dephosphorylation of plasma membrane H^+^-ATPases. In their active phosphorylated form, H^+^-ATPases bring about cell wall acidification, resulting in cell elongation [10]. Two recent reports demonstrate that auxin-induced growth requires SAUR proteins and occurs by activation of H^+^-ATPases, and that overexpression of SAUR proteins is sufficient to activate the H^+^-ATPases independent of auxin [6, 11]. Thus, SAURs act downstream of auxin and are also able to induce growth when activated by other factors than auxin.

Although *SAURs* were first discovered as auxin-induced genes, more recent reports show that their regulation is much more diverse and does not only depend on auxin, but on a variety of factors including different hormones, light response factors and other transcription factors. Of the Arabidopsis *SAUR* genes, approximately two-third can respond to auxin in certain tissues and functions downstream of the Auxin Response Factors ARF5, ARF6, ARF7, ARF8 or ARF19 [12–19]. However, several *SAUR*s are also regulated by other hormones, such as abscisic acid (ABA), ethylene, gibberellic acid (GA) and brassinosteroids (BR) [7, 20–24]. In addition, *SAUR*s have been identified as direct targets of the light response transcription factor PHYTOCHROME INTERACTING FACTOR 3 (PIF3) and PIF4 [9, 22], the BR response factor BRASSINAZOLE RESISTANT 1 (BZR1) [22], and the MADS-domain TF FRUITFULL (FUL) [3]. Thus, the picture emerges that SAUR proteins can generally fulfil growth-promoting functions by activating H^+^-ATPases, but that activation and/or repression of the different *SAUR* genes depends on combinations of cis-elements that are specific for the different genes.

The growth-inducing capacity of SAURs has been best characterized in seedlings. Sun and co-authors recently showed that SAURs are both responsible for hypocotyl elongation in the dark and for the expansion of cotyledons upon transfer of seedlings to the light [9]. In both tissues, a large group of *SAUR* genes responded to light, either being repressed in hypocotyls via the auxin pathway or induced in cotyledons by PIFs [9]. As it turns out that multiple *SAUR* genes can simultaneously respond to particular growth-inducing signals, the function of *SAURs* in different tissues is very difficult to assess by knock-out studies. Subtle growth responses have been detected in seedlings upon double knock-out [9], but have not been reported from other tissues because these are less easy to monitor precisely. However, recent reports describing specific upregulation of *SAUR* genes at the abaxial side of the stem in Arabidopsis [3] and sunflower [25], indicate that SAURs can also be responsible for dynamic growth responses in other tissues. This suggests, together with the fact that various other tissues need to grow rapidly by cell elongation, that SAURs mediate growth responses throughout the plant. However, which SAURs function where and how important their role is in different tissues remains unclear.

To further unravel the role of SAURs throughout the plant, we characterized a clade of eight *SAUR* genes in Arabidopsis: *SAUR8*, *SAUR9*, *SAUR10*, *SAUR12*, *SAUR16*, *SAUR50*, *SAUR51* and *SAUR54*. We performed overexpression and knock-out experiments, investigated their expression patterns and studied their responses to different hormone applications. In addition, we carried out in silico analyses using the regulatory regions of all Arabidopsis *SAUR* genes to better predict their different roles in plant growth dynamics.

## Results

### *SAUR8/10/16* overexpression lines show similar growth-related phenotypes

**Fig. 1:**
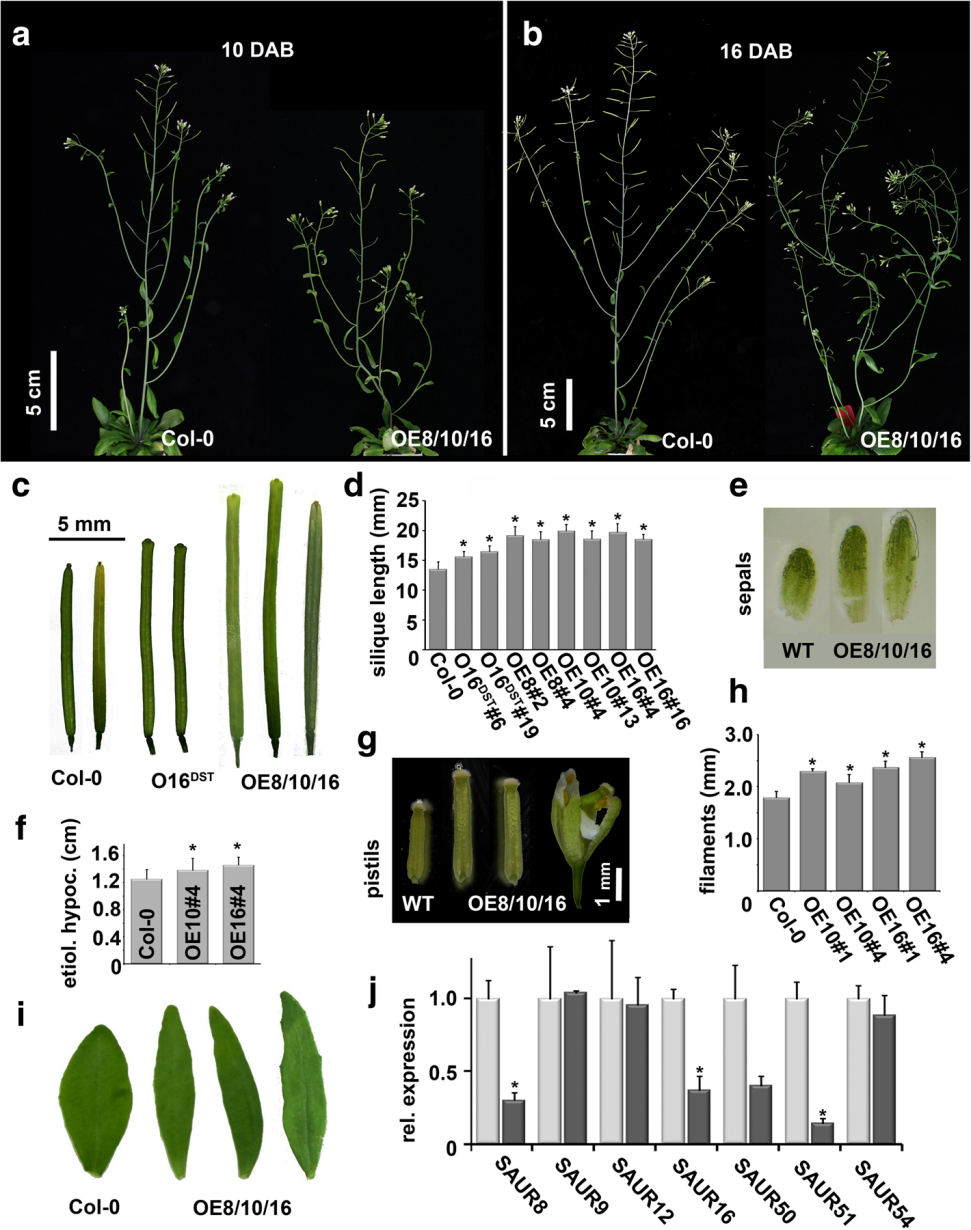
Overexpression phenotypes of the *SAUR8*, *SAUR10* and *SAUR16* overexpression lines. **a**-**b** Whole-plant phenotypes of representative Col-0 and *35S:SAUR8/10/16* overexpression lines. Because the phenotypes from the three different constructs were comparable (there is more variation between lines from the same construct than between constructs), only two representative plants are displayed here. The overexpression lines display wavy main stems and side branches, show reduced fertility due to the long pistil size, and have an irregular phyllotaxy. **a** Col-0 and *35S:SAUR10#4* at 10 days after bolting (DAB). **b** Col-0 and *35S:SAUR8#2* at 16 days after bolting (DAB). **c** The siliques of the different overexpression lines are significantly longer than wild-type siliques and have ‘bumpy shoulders’. O16^DST^ = *35S:SAUR16* including the DST region; OE8/10/16 = *35S:SAUR8/10/16* all OE8/10/16 lines showed a similar silique phenotype, a few representative siliques are shown. **d** Quantification of silique length in the different overexpression lines. From each construct, The lines with the longest siliques were selected for quantification. O16^DST^#6 = 35S:SAUR16DST line 6; OE8#2 = 35S:SAUR8 line 2; etc. **e**-**i** Various organs are longer in the *SAUR* overexpression lines. The phenotypes from the different constructs were very similar, with more variation between the lines of the same construct than between different constructs, and are therefore noted as OE8/10/16 (35S:SAUR8, 35S:SAUR10 or 35S:SAUR16). The phenotypes from the strongest lines are shown. **e** Length of the sepals of stage 13 flowers. **f** Length of etiolated hypocotyls 7 days after stratification. **g** Length of the pistils of stage 13 flowers. **h** Length of the filaments of stage 13 flowers. **i** Cauline leaf length and shape. **j** Expression of the *SAUR10*-clade genes in the stem of *35S:SAUR10* lines, relative to the expression in wild-type stems. Significant differences (T-test, *p* < 0.05) are indicated with an asterisk

We previously identified *SAUR10* as a target of the MADS-domain factor FRUITFULL during stem growth [3]. *SAUR10* belongs to a clade of eight closely related Arabidopsis *SAUR* genes, consisting of *SAUR8*, *SAUR9*, *SAUR10*, *SAUR12*, *SAUR16*, *SAUR50*, *SAUR51* and *SAUR54*, hereafter referred to as the *SAUR10*-clade. Based on their protein sequences, the members of the clade can be grouped in four pairs of two paralogs each (SAUR8/SAUR50, SAUR12/SAUR16, SAUR51/SAUR54 and SAUR9/SAUR10) (see Additional file 1: Figure S1), which have highly similar sequences, ranging from 64% identity for the most distant sequences (SAUR9/SAUR54) to 93% identity for the most similar sequences (SAUR12/SAUR16). Despite their close homology, the genes respond differently to hormone application as deduced from previous publications (see Additional file 2: Table S1) [8, 12–14, 18, 19, 26]. To obtain more information about the putative functions of all members of the SAUR10-clade, and to increase the general understanding of SAUR function, we characterized all eight genes. We started with determining the protein functions of different SAURs from the SAUR10-clade, and generated overexpression constructs for three members of the clade with relatively little sequence identity, *SAUR8*, *SAUR10* (also described in [3]) and *SAUR16* (between 72% and 75% identity). Because the DST region (for DownSTream element) in the 3’UTR of *SAUR* genes has been shown to be involved in transcript stability [27], we generated *SAUR16* constructs excluding and including the DST region (*35S:SAUR16* and *35S:SAUR16DST*, respectively) to test whether this affected transcript accumulation. For all constructs, we generated approximately one hundred transgenic lines. There was a clear difference in the frequency of aberrant phenotypes between the *35S:SAUR16DST* lines and the *35S:SAUR8/10/16* lines. From the one hundred *35S:SAUR16DST* lines, only nine showed a mild phenotype, which was restricted to the siliques (see below). On the other hand, over 50% of the *35S:SAUR8*, *35S:SAUR10* and *35S:SAUR16* lines showed mild to severe phenotypic aberrations. The *35S:SAUR8*, *35S:SAUR10* and *35S:SAUR16* lines all showed similar growth-related phenotypes, including longer etiolated hypocotyls, filaments, sepals, cauline leaves and a wavy stem (Fig. 1). The pistils were also markedly longer and often had to be hand-pollinated, as the anthers did not reach the stigma. Efficient fertilization resulted in very long siliques (Fig. 1c and d). All phenotypic aberrations were shared among the lines of the three overexpression constructs, construct-specific phenotypes were not observed. The phenotypic characterization shown in Fig. 1 was performed with the strongest lines. In contrast to the *35S:SAUR16* lines, the *35S:SAUR16DST* phenotype was limited to aberrantly shaped ‘bumpy’ siliques that were longer than the wild type (Fig. 1c and d). These data indicate that the *35S:SAUR16DST* construct is much less effective than the *35S:SAUR16* construct, probably because the presence of the DST region causes transcript instability and thus much lower *SAUR16* levels. Our results show that the different SAUR10-clade proteins can influence growth in various parts of the plant in a similar manner.

### Single and double T-DNA mutant lines do not exhibit mutant phenotypes

To investigate the role of the different *SAUR10*-clade genes further, we searched for available T-DNA insertion mutants. We identified lines for each of the eight genes (see Additional file 3: Figure S2), but could not confirm the presence of a T-DNA insertion in *saur50* (SALK_022758) and *saur10–1* (FLAG_590D09). In addition, four other lines could be confirmed by genotyping, but had the T-DNA insertion upstream of the first exon or showed only partial transcript reduction (*saur10–2*, SM_3_1724; *saur9–2*, SALK_054423; *saur12*, SALK_09008C0; *saur54*, SALK_054420). The remaining lines that showed a clear transcript down-regulation (*saur8*, SALK_003272; *saur9*, SALK_054423; *saur16*, SALK_003390; *saur51*, FLAG_496D01) exhibited wild-type phenotypes throughout plant development. We generated several double and triple mutant combinations to further search for mutant phenotypes (see Additional file 3: Figure S2), but observed only wild-type phenotypes upon inspection of seedling growth, rosette and cauline leaf shape, plant architecture, flower phenotype and pistil/silique size. The analysis of a *saur50saur16* double mutant generated using the CRISPR/Cas9 system recently revealed mild growth retardation in seedlings [9], confirming the role of *SAUR16* and *SAUR50* in cell elongation. The lack of mutant phenotypes in our analyses is probably largely due to the high level of redundancy amongst the *SAUR*s and lack of decent knock-out lines. However, it is possible that mild phenotypes can be detected when very detailed measurements or specific assays (response to light/hormones) are performed.

In addition, phenotypic effects of *SAUR* knock-outs may be levelled out by feedback mechanisms that allow compensation for the knock-out of certain *SAUR*s by up-regulation of others, resulting in a null effect on the phenotype. To determine whether *SAUR* expression is responsive to up- or down-regulation of other *SAUR*s, we tested the expression of all *SAUR10*-clade genes in stems of the *35S:SAUR10* overexpression lines, and found that *SAUR8*, *SAUR16*, *SAUR50* and *SAUR51* have a clearly reduced expression in response to *SAUR10* overexpression (Fig. 1j), suggesting the existence of SAUR feedback loops that can compensate for de-regulated *SAUR* expression.

### *SAUR10*-clade genes have diverse expression patterns

**Fig. 2:**
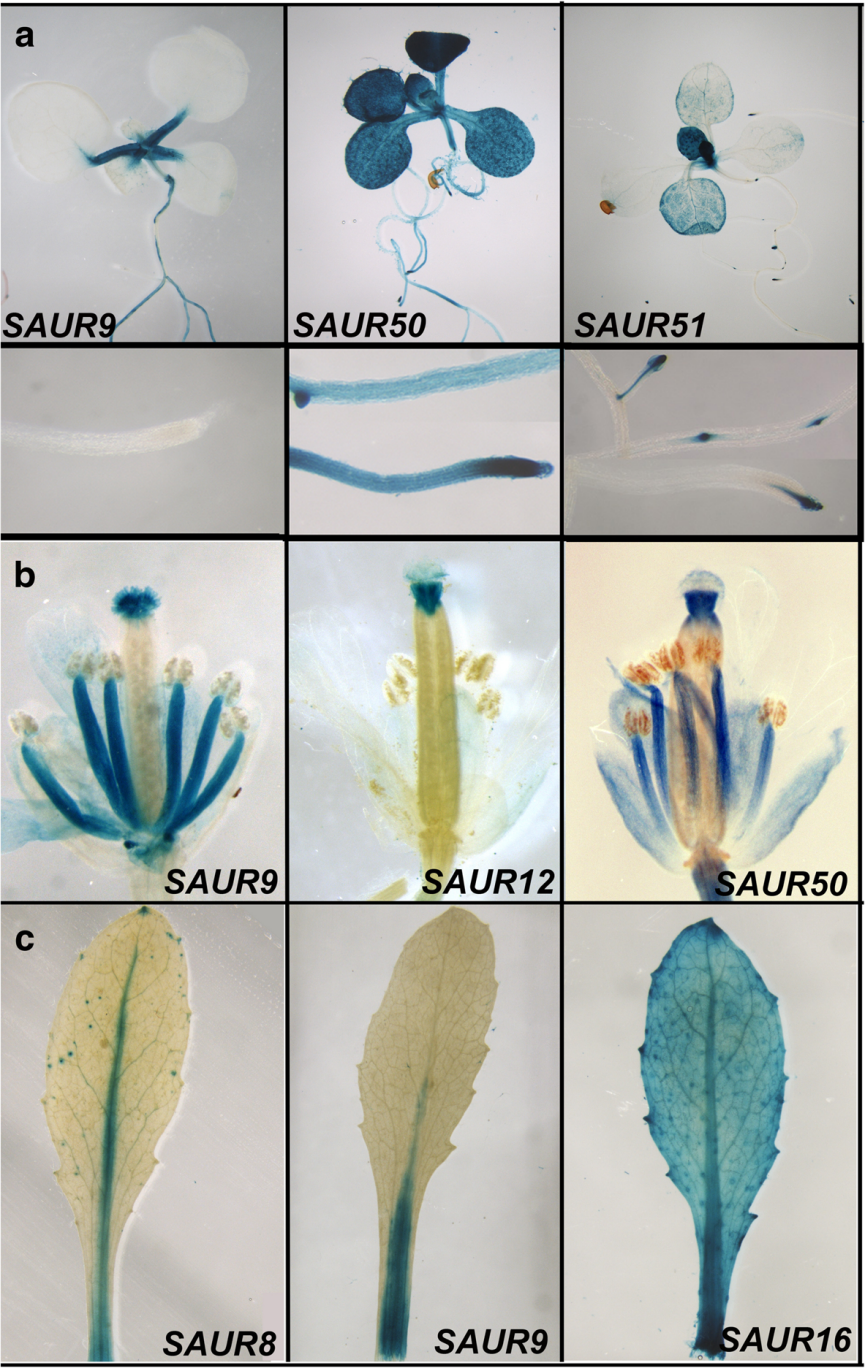
The *SAUR10*-clade genes exhibit diverse expression patterns. Selection of pictures showing the expression in different tissues. GUS staining was performed overnight. The complete sets of pictures are shown in Additional file 4: Figure S3. **a** Expression of *pSAUR9:GUS*, *pSAUR50:GUS* and *pSAUR51:GUS* in 14 day-old seedlings. The lower panels show a magnification of the staining patterns in the seedling root. **b** Expression of *pSAUR9:GUS*, *pSAUR12:GUS* and *pSAUR50:GUS* in open flowers. **c** Expression of *pSAUR8:GUS*, *pSAUR9:GUS* and *pSAUR16:GUS* in mature cauline leaves

The protein functions of the different SAURs appear to be similar, but their roles during plant growth and development may be very different, depending on variation in their regulatory regions that can bring about specific expression patterns and responses to upstream factors. To investigate the expression patterns of the eight *SAUR10*-clade genes, we generated promoter:reporter constructs for each gene containing approximately 3 kb upstream sequence fused to the β-glucuronidase (*GUS*) reporter gene and compared the observed expression patterns in different parts of the plant (Fig. 2 and Additional file 4: Figure S3). For each construct, approximately 20 lines were raised, of which two strong lines were characterized in detail. This yielded consistent patterns for all lines generated with the same reporter construct, except for *pSAUR54:GUS*, of which the lines displayed some variability in the presence or absence of signal in different tissues. Interestingly, the GUS expression patterns for the different constructs were highly diverse, suggesting that the *SAUR*s of the *SAUR10*-clade fulfil different functions depending on the tissues where they are expressed. We observed for example distinct expression of *SAUR54* in trichomes, *SAUR51* in primordia, *SAUR9* in petioles and *SAUR12* in styles. However, in most tissues, two or more of the *SAUR10*-clade genes were found to be co-expressed, suggesting redundant functions for the *SAUR10*-clade genes.

Some of the *SAUR* genes, such as *SAUR16* and *SAUR50*, were expressed in a wide range of tissues. GUS staining for these reporters could be observed in the cotyledons, rosette leaves, cauline leaves, flower and silique. Other *SAURs* showed a more specific expression pattern (Fig. 2 and Additional file 4: Figure S3). *pSAUR9:GUS* for example, exhibited specific expression in the midvein of the petiole and leaf blade, and was in the flower only expressed in the filaments and stigma papillae. *pSAUR12:GUS* showed a highly specific staining in the flower, with only expression in the style of stage 13–16 flowers. The expression pattern of *pSAUR8:GUS* was in leaves restricted to the midvein and petiole, similar to *SAUR10* and *SAUR9*, while the pattern in the mature flower resembled that of *pSAUR16:GUS*. Finally, *SAUR51* and *SAUR54* showed again different expression patterns. They were hardly expressed in expanded rosette leaves or cauline leaves, but *pSAUR51:GUS* was highly active in root primordia, leaf primordia and flower primordia, while *pSAUR54:GUS* was the only *SAUR10*-clade gene that showed high expression in the trichomes of emerging leaves.

### *SAUR10*-clade promoters contain multiple TF binding sites

The diverse expression patterns of the *SAUR10*-clade genes throughout plant development are probably a result of regulation by different TFs. *SAURs* have been identified as targets of different TFs involved in growth and development, such as the MADS-domain TFs SEPALLATA3 (SEP3) [28], APETALA1 (AP1) [29], and FUL [3], and the TCP (TEOSINTE BRANCHED1 /CYCLOIDEA/PROLIFERATING CELL FACTOR1) family protein TCP20 [30]. To get more insight into the TFs that can directly regulate the *SAUR10*-clade genes, we searched the plant DHS database (http://plantdhs.org/) [31], which contains ChIP-seq data from 26 different TFs, for occurrences of TF binding sites in the *SAUR10*-clade promoters (see Table 1). We found that the *SAUR*s were ubiquitously present in the ChIP-seq data of different TFs, in particular of APETALA2 (AP2), and the MADS-domain TFs AGL15, AP1 and SEP3, which are involved in flowering and floral organ development, and the PSEUDO-RESPONSE REGULATORS (PRR) PRR5 and PRR7, which function in circadian clock-controlled processes. Thus, the expression of *SAUR10*-clade genes in the inflorescences and floral organs is probably largely controlled by MADS-domain TFs and AP2, and the amplitude of this expression is expected to be regulated by the circadian clock. Interestingly, the widely expressed *SAUR16* and *SAUR50* genes also show the highest number of TF binding events. More specific binding by certain TFs could also be identified, such as the binding of FAR-RED ELONGATED HYPOCOTYLS 3 (FHY3), involved in the phytochrome pathway, in the upstream region of *SAUR16*, and PISTILLATA (PI), specifically involved in petal and stamen development, upstream of the *SAUR16* and *SAUR51* coding regions. These results show that *SAURs* are indeed common targets of TFs controlling plant development and growth.

**Table 1:** Transcription factor binding events in the upstream regions of *SAUR10*-clade genes

	*SAUR8*	*SAUR9*	*SAUR10*	*SAUR12*	*SAUR16*	*SAUR50*	*SAUR51*	*SAUR54*
*AGL15*		+	+		+	+	+	
*AP1*	+	+	+	+	+	+	+	
*AP2*		+		+	+	+	+	
*FHY3*					+			
*LFY*					+	+		+
*PI*					+		+	
*PIF3*						+		
*PIF4*			+	+	+	+		
*PIF5*					+	+		
*PRR5*	+	+	+	+	+	+	+	
*PRR7*			+	+	+	+		
*SEP3*		+	+	+	+	+	+	+
*SOC1*		+	+					

The DHS database (http://plantdhs.org/) was searched for the occurrence of binding sites in ChIP-seq data from 26 TFs (AGL15, AP1, AP2, AP3, BES1, EIN3, ERF115, FHY3, FLC, FLM, FUS3, GL1, GL3, GTL1, LFY, PI, PIF3, PIF4, PIF5, PRR5, PRR7, SEP3, SMZ, SOC1, TOC1, WUS). 3000 bp of upstream region was used for the search. Grey shading indicates a binding site in this region

### *SAUR10-*clade genes respond differently to hormones

**Fig. 3:**
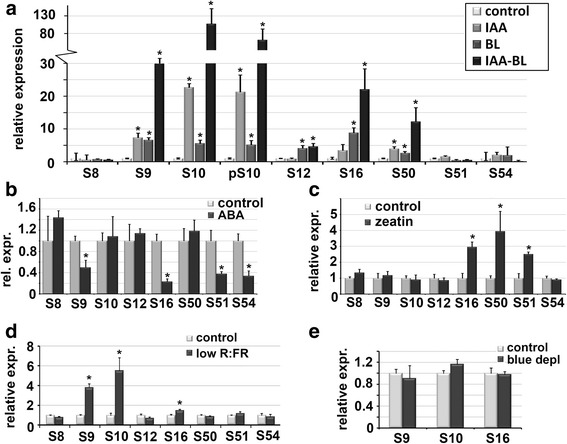
*SAUR10*-clade genes respond differently to application of various hormones. **a** Expression of the *SAUR10*-clade genes in seedlings after a 4 h treatment with auxin, brassinosteroids or a combination of both. The numbers on the x-axis stand for the different *SAUR* genes, p10 stands for *pSAUR10:GUS*, of which the *GUS* expression was monitored. **b** Expression of the *SAUR10*-clade genes in seedlings after 4 h of ABA treatment relative to the expression in mock treated seedlings. **c** Expression of *SAUR10*-clade genes in seedlings after 4 h of zeatin treatment relative to the expression in mock treated seedlings. **d** Expression of the *SAUR10*-clade genes in seedlings grown for 4 h under reduced R:FR conditions and harvested directly after, compared to seedlings grown for 4 h under control conditions. **e** Expression of *SAUR9*, *SAUR10* and *SAUR16* in seedlings after 4 h growth under depleted blue-light conditions. The error bars represent the SE based on three or four biological replicas. Significant differences (t-test, *p* < 0.05) are indicated with an asterisk

High-throughput transcriptome analyses have revealed that different sets of *SAUR* genes can respond to various hormonal cues such as auxin, BR and ABA (see [8] and Additional file 2: Table S1 for an overview). However, some *SAUR* genes do not appear to respond to any of these hormones. To investigate the response to hormones in more detail for the *SAUR10*-clade genes, we performed several hormone induction experiments using 14 day-old seedlings. *SAUR10* was highly induced after 4 h of incubation in liquid medium supplemented with auxin (5 μM IAA [3, 14]), and also *SAUR16, SAUR9* and *SAUR50* showed a significant increase in expression after application of auxin (Fig. 3a). We tested the response to various other hormones and demonstrate that the same genes also respond to brassinosteroids (BR) (Fig. 3a), as has been reported before for some *SAUR*s, including *SAUR10* [3, 21, 22, 24]. *SAUR12* was also 4-fold induced in response to BR application. When applying both IAA and BR, *SAUR9* and *SAUR10* showed a strong synergistic response with transcript levels rising to 100 times the level of control conditions, and also *SAUR16* and *SAUR50* showed an enhanced response. This shows that many auxin-regulated *SAURs* are also regulated by BR in a combinatorial way. However, the fact that *SAUR12* only responds to BR indicates that the responses can also be uncoupled. To investigate the dynamics of IAA-BR induction, we also tested the decline of *SAUR9* and *SAUR10* expression after removal of the induction medium, and found that transcript levels dropped within one hour (Additional file 5: Figure S4), illustrating the rapid breakdown of *SAUR* transcript and thereby the dynamic nature of the growth response.

In contrast to auxin and BR, the abiotic stress-related hormone ABA can affect growth in a negative way, which could be regulated by repression of *SAUR* genes as well. Indeed, the abiotic stress responsive TFs AZF1 (Arabidopsis Zinc-Finger protein 1) and AZF2, which down-regulate many ABA- and Osmotic Stress-Repressive Genes, also repress a number of *SAURs* [20]. We tested the expression of the *SAUR10*-clade genes after 4 h of 100 μM ABA treatment and found a significant down-regulation of *SAUR9*, *SAUR16*, *SAUR51* and *SAUR54* (Fig. 3b), supporting the role of SAURs in ABA-induced growth retardation. We also investigated the response to cytokinin (1 μM Zeatin), and found that *SAUR16*, *SAUR50* and *SAUR51* were upregulated in response to cytokinin (Fig. 3c), which for *SAUR50* and *SAUR51* nicely correlates with their expression in root tips (see Fig. 2). Treatment with GA did not result in major changes in transcript levels, but *SAUR9* showed a 2-fold decrease in expression, while *SAUR54* transcript levels were mildly upregulated (Additional file 6: Figure S5). In conclusion, the hormone application experiments show that the *SAUR10*-clade genes respond very differently to various hormones.

### The reporter-gene patterns correlate with hormone-induced expression

Several *SAUR*s of the *SAUR10*-clade show dark-induced expression in the hypocotyls (Additional file 4: Figure S3). Especially *SAUR9*, *SAUR10* and *SAUR16*, of which the reporters do not show any GUS signal in light-grown hypocotyls, show a very distinct signal in dark-grown hypocotyls. This corresponds with the IAA-BR experiments, which show that *SAUR9*, *SAUR10* and *SAUR16* have the highest response to these hormones. These data suggest that the response to auxin and BR occurs at the promoter level, as has been reported for *SAUR15* [21]. In addition, *GUS* expression in the *pSAUR10:GUS* line could be induced to similar levels as *SAUR10* itself after combined IAA-BR treatment (Fig. 3a; [3]), indicating that all elements necessary for the induction of *SAUR10* by IAA and BR are present in the promoter fragment. To investigate this further, we also tested whether IAA/BR induced *SAUR* expression occurs ectopically, or at the location of the endogenous expression. *pSAUR:GUS* seedlings of the highest responders, *SAUR9*, *SAUR10* and *SAUR16*, were treated with Mock or IAA/BR as before, and subsequently stained for GUS activity (Additional file 7: Figure S6). Increased GUS signal was only observed at the location of endogenous expression (Additional file 4: Figure S3 and Additional file 7: Figure S6), suggesting that IAA/BR can only enhance promoter activity in tissues where the genes are already active.

In addition to hypocotyl growth, also stamen filament elongation has been shown to depend on auxin and BR [15, 32, 33]. Interestingly, the *SAUR10*-clade genes that show a clear response to auxin and BR, *SAUR9*, *SAUR10*, *SAUR16* and *SAUR50*, all have a high expression during late stages of filament elongation (Fig. 2 and Additional file 4: Figure S3), and have been identified as direct targets of ARF6/ARF8 and BZR1 (Additional file 2: Table S1; [15, 22]). These data strengthen the idea that *SAUR9*, *SAUR10*, *SAUR16* and *SAUR50* may function downstream of auxin and BR to regulate growth of, for example, the stamen filaments.

### SAUR9, SAUR10 and SAUR16 are upregulated under reduced R:FR light

In response to shaded conditions, plants adjust their growth program and exhibit an altered morphology including stem elongation, petiole expansion and hyponastic leaf movement, collectively referred to as the Shade Avoidance Syndrome (SAS) [34]. The SAS response is regulated by both auxin and BR in response to either reduced R:FR light ratios or depletion of blue light, and could thus be regulated by *SAUR* genes as well [35, 36]. Recently, the expression of *SAUR10* indeed revealed to be upregulated in response to low R:FR light conditions [3]. We tested the expression levels of all *SAUR10*-clade genes by transferring 14 day old seedlings to low R:FR light conditions and compared the expression of the genes with seedlings from the control condition. *SAUR9* and *SAUR10* showed a marked increase of expression after 4 h of low R:FR light, while a mild, but significant increase could be detected for *SAUR16*. The other *SAURs* of the clade did not respond to the changed light conditions (Fig. 3d). We also tested whether *SAUR9*, *SAUR10* or *SAUR16* responded to blue light depletion, as the response to this light condition has been shown to be related to a combination of auxin and brassinosteroids [35], but the expression levels were similar to control conditions (Fig. 3e). The response of *SAUR9* to simulated shade conditions is in particular interesting, because its specific expression in the leaf petioles could be well linked to a role in the SAS response.

### The presence of AuxRE and BZR motifs in the *SAUR* promoters correlates with their auxin responsiveness

The results of the hormone induction studies and expression analyses reveal that *SAUR* genes have specific expression patterns and respond differently to application of various hormones. Based on our results and the high-throughput studies published before, *SAUR* genes can be divided into two groups: I) *SAUR* genes that are induced in response to auxin application, II) *SAUR* genes that do not respond to auxin application (see Additional file 2: Table S2). Of the *SAUR10*-clade genes, *SAUR9*, *SAUR10*, *SAUR16* and *SAUR50* belong to the auxin-induced group (Class I), while *SAUR8*, SAUR12, and *SAUR54* are not regulated by auxin (Class II). The auxin-inducibility of the class I *SAUR10*-clade genes can be regulated by ARFs, which is illustrated by the fact that *SAUR10* is down-regulated in *arf5* mutants [17], *SAUR9* in both *arf5* and *arf7/19* mutants [16, 17], and *SAUR10*, *SAUR16* and *SAUR50* have been identified as direct ARF targets in ChIP-seq experiments [22]. In our assay, *SAUR51* did not respond to auxin in seedlings, but a previous study has shown that *SAUR51* is induced by auxin in roots [12], and we therefore added *SAUR51* to the class I genes. We summarized the results from previous studies in Additional file 2: Table S1, and found that 50 *SAUR* genes belong to Class I (induced by auxin), while 20 genes did not respond to auxin in any of the experiments and were grouped in Class II [12–14, 19] (see Additional file 2: Table S2). This suggests that, despite their name, this latter group of *SAUR* genes is regulated by other factors than auxin.

To investigate whether these groups could also be distinguished based on the presence of certain cis-elements in their regulatory regions, we searched for auxin-related cis-elements in the 3 kb upstream regions of all *SAUR*s. ARFs bind to AuxRE motifs (TGTCTC/GAGACA [37] or TGTCGG/CCGACA [38], which could be identified in almost all *SAUR* regulatory sequences; there was no significant difference between Class I and Class II promoters, nor between allSAURs (Class I and II together) and a random control group in the abundance of the single AuxRE motifs. However, ARFs bind *in planta* usually as a homodimer to an everted repeat of two AuxRE elements, separated by a particular spacing [37, 38]. The consensus for this combined binding site can be noted as TGTC[x]_11-13_GACA, where TGTC and GACA represent the core AuxRE binding sites, while [x]_11–13_ denotes the number of nucleotides in between. We tested this motif with the three different spacing variants, and found a distinct over-representation of this motif in the Class I (auxin-induced) promoters. The fraction of Class I promoters that contained the motif was 0.22 (11 out of 50, among which *SAUR10* and *SAUR51*), while this fraction was 0 for Class II promoters (0 out of 20) and 0.07 for the control group. Both the difference between Class I and Class II promoters (p~0.02) and between Class I and control group promoters (p~0.02) was significant. Variations on this combined consensus motif have been published (e.g. GGTC[x]_11_GACA [38]) and also the spacing can be more flexible for certain ARFs [38], indicating that a larger fraction of *SAUR*s might be identified with the combined AuxRE motif when testing a more relaxed motif. For example, the *SAUR16* promoter contains a GGTC[x]_9_GACA motif, which can possibly be bound by ARF5 [38]. However, testing more relaxed motifs also increased the occurrence of combined AuxRE motifs in Class II genes. Interestingly, also the BZR1 binding motif (CACGTG) was over-represented in the Class I promoters in comparison with the Class II promoters (10/50 vs 0/20, p~0.03), and was identified in the promoters of *SAUR10*, *SAUR12* and *SAUR16*, but not in the non-BR responsive *SAUR10*-clade *SAURs*, confirming the link between auxin and BR regulation of certain *SAURs* [21, 22]. These in silico data support the finding that not all *SAUR*s can be induced by auxin, and suggest that the lack of auxin-responsiveness in the Class II *SAUR* genes is caused by the absence of combined AuxRE elements, which is accompanied by a lower fraction of BZR1 motifs.

### De novo motif search reveals a PIF5 binding site containing motif

**Fig. 4:**
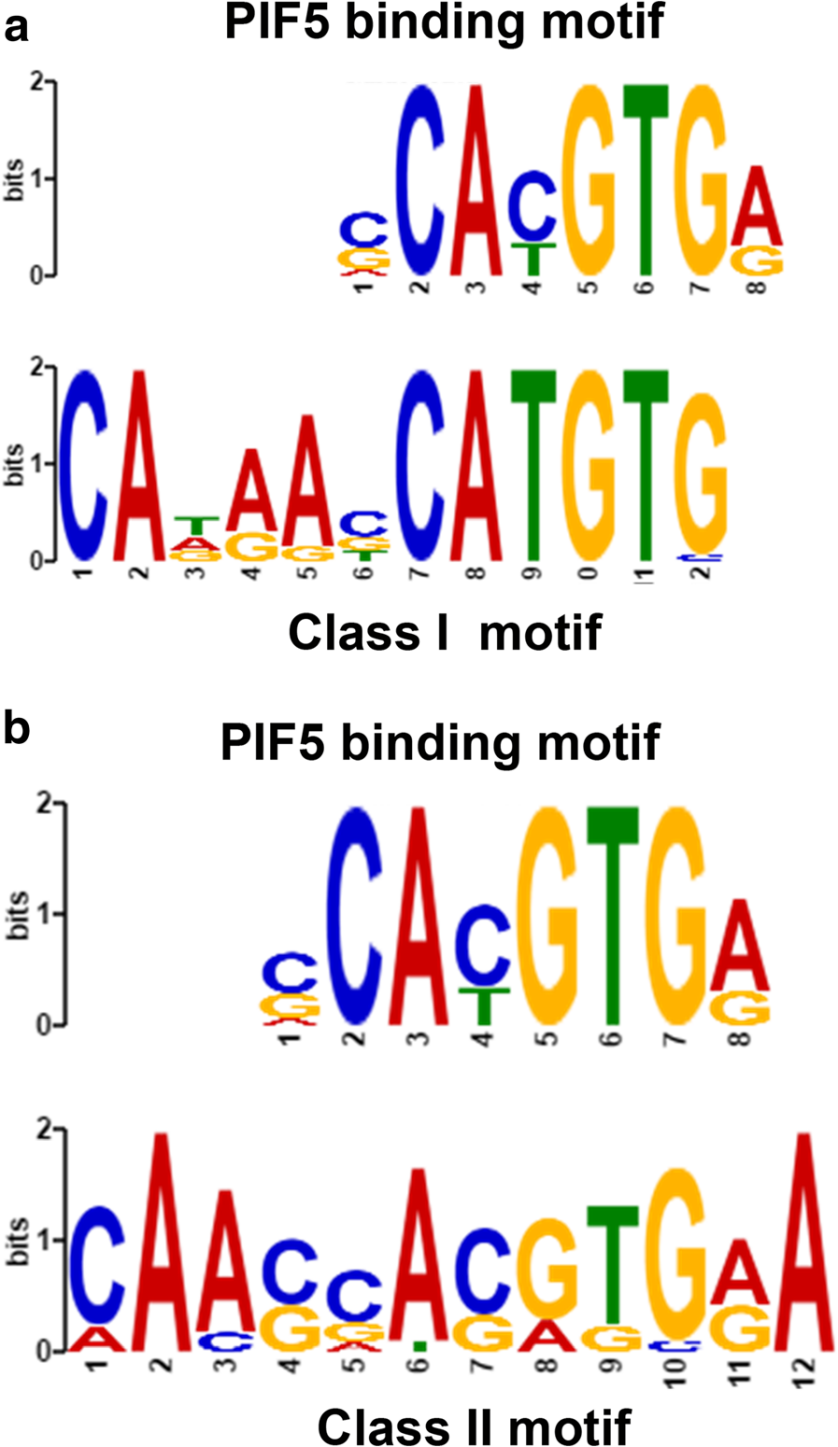
Motifs identified in the Class I and Class II *SAUR* promoters both match the PIF5 binding site. **a** The motif identified in the Class I promoters is significantly over-represented compared to Class II and random promoters. Upper panel: PIF5 binding motif (MA0562.1), Lower panel: Identified Class I motif. **b** The motif identified in Class II promoters is not significantly enriched compared to Class I and random promoters. Upper panel: PIF5 binding motif (MA0562.1), Lower panel: Identified Class II motif

To determine whether other transcription factors could be identified as common regulators of *SAUR* genes, we performed a de novo motif search. This revealed one motif specific for the Class I *SAUR* promoters, and another, slightly different motif for the Class II *SAUR* promoters (see Fig. 4). Using TOMTOM, we checked for the occurrence of known binding sites in both motifs and found for both a single match with the PIF5 motif. Hence, both in the set of *SAURs* regulated by auxin and those not regulated by auxin, a PIF5-like motif was identified, although they are somewhat different from each other and extended in comparison to the known canonical PIF5 binding site. To further investigate this, a consensus version of these two motifs was tested for its occurrence in both sets. The motif CAxxxxCATGTG (consensus version of the motif shown in Fig. 4a) is present in 28 out of 50 auxin-induced *SAURs* (among which *SAUR10*) and only in 2 out of 20 Class II *SAURs*; the latter being similar to the occurrence of 56 in 500 randomly chosen promoters. The difference between regulated and not-regulated sequences is significant (*p* = 0.0003). Hence, the PIF5-like motif CAxxxxCATGTG is clearly more often present in the Class I *SAUR*s than in Class II *SAUR*s and control promoters. The alternative CA[AC]xxAxGTG (consensus version of the motif shown in Fig. 4b) does not show significant differences in occurrence between the Class I *SAURs*, Class II *SAURs* or random background.

All PIFs bind preferentially to G-boxes (5′-CACGTG-3′) [39], and PIF5 also binds to the Class I motif 5′-CATGTG-3′ [40]. Core G-boxes (PIF4/5 binding sites) are very abundant and present in all *SAUR10*-clade genes. In accordance with this, many *SAURs* have been identified as direct targets of PIF4, including *SAUR9*, *SAUR10*, *SAUR12*, *SAUR16*, *SAUR50* and *SAUR51* [22], as well as of PIF5 [40] (see Additional file 2: Table S1). Whether PIF5 plays a specific role in the regulation of Class I SAURs remains to be investigated. The down-regulation of *SAUR9*, *SAUR10* and *SAUR16* in hypocotyls of the *pifq* quadruple mutant (*pif1*, *pif3*, *pif4* and *pif5*) [9] at least shows that the light-responsive *SAUR10*-clade *SAURs* are regulated by PIFs. Moreover, in a study by Hornitschek et al. [40], *SAUR10* and *SAUR16* are the only *SAURs* that are significantly down-regulated in *pif4pif5* seedlings, and only *SAUR10* is down-regulated in *pif5* single mutant seedlings. It would be interesting to study the role of the CAxxxxCATGTG motif in the *SAUR10* promoter for its PIF5-dependent regulation. The identification of a longer motif here may point to the binding of a second transcription factor in close proximity to the PIF. Our in silico analysis underlines the importance of the PIFs for *SAUR* regulation in general, but also reveals differences between the group of auxin-induced *SAUR*s and the group of non-auxin regulated *SAUR*s. Except for some enrichment of A/T-rich stretches in the *SAUR* promoter sequences, no other binding sites were identified that were significantly over-represented in the *SAUR* promoters compared to the control promoters.

## Discussion

### SAURs allow plants to fine tune growth in various tissues

We show here that the eight *SAUR10*-clade genes are likely to have similar protein functions, but exhibit clearly distinct expression patterns. We therefore expect that all SAUR10-clade proteins are able to promote cell elongation by inducing cell wall acidification as was shown for SAUR9 and SAUR50 [9, 10], while the different cis-element combinations in the upstream regions of each gene allow a very specific activity. In line with previous results, we found some of the *SAUR*s to be highly responsive to hormones, while others did not respond at all. Comparison of our data with previously published lists of auxin-responsive genes [12–14, 19] revealed that the response to auxin is quite consistent in different experiments, and that *SAUR9*, *SAUR10*, *SAUR16* and *SAUR50* generally respond to auxin, while *SAUR8*, *SAUR12*, and *SAUR54* never do. This suggests that part of the *Arabidopsis SAUR* genes does not have the ability to respond to auxin and may regulate growth in an auxin-independent manner, although we cannot exclude that some of these genes, such as *SAUR51*, are specifically induced by auxin in particular tissues. The auxin insensitivity of the non-responsive genes was supported by our in silico data, which revealed an over-representation of the combined AuxRE element in the group of auxin-induced genes only. Nevertheless, genes in the non-induced group can still be important for cell elongation, as the overexpression of SAUR proteins is sufficient to activate the H^+^-ATPases independent of auxin [6, 11], and several of these *SAUR* genes show distinct expression patterns. For example, *SAUR8* is not induced by auxin or brassinosteroids, but the *35S:SAUR8* overexpression line has a clear cell elongation phenotype, while the *pSAUR8*:GUS reporter line shows distinct expression in various tissues. Similarly, *SAUR51* is specifically expressed in the root tips and expanding leaves (Fig. 2 and Additional file 4: Figure S3). The specific expression of some *SAURs* outside cell elongation domains, such as the expression of *SAUR51* in the root meristem, is interesting and may point to alternative functions for SAURs besides cell elongation.

Thus, the picture emerges that *SAUR* genes are generally important for cell elongation, but are each regulated by a specific set of upstream factors, allowing the plant to fine tune growth in a variety of tissues in response to internal and external signals. Our data also show however, that the *SAUR10*-clade *SAURs* have overlapping expression patterns, in agreement with the data of Sun et al. [9], which identified groups of *SAUR* genes that responded similarly to light conditions in hypocotyls and cotyledons. This suggests that the growth response in each tissue is regulated by a cluster of similarly regulated *SAURs*, rather than by single genes. To identify *saur* mutant phenotypes, higher order mutants may therefore better be generated based on the expression pattern of the genes than on their homology.

### Combinatorial response of *SAUR*s to auxin, BR and light conditions

That *SAUR*s can respond to both auxin and brassinosteroids has been previously reported [3, 19], and Walcher and Nemhauser [21] showed that the auxin response of *SAUR15* depends on a functional brassinosteroid pathway. We show here that also *SAUR9*, *SAUR10*, *SAUR16* and *SAUR50* expression can be induced to a much higher level by a combination of auxin and BR, indicating that a synergistic response may be common to genes of the *SAUR* family. This is also supported by our finding that AuxRE and BZR binding motifs are highly over-represented in the group of auxin-induced genes compared to the non-regulated genes. Interestingly, we also found the PIF5 binding site over-represented in the auxin-induced class only, suggesting that PIF-directed regulation may also occur in concert with auxin and BR response factors. In agreement with a general interdependency of the three factors, Oh et al. discovered that ARF6, PIF4 and BZR1 can physically interact with each other to cooperatively regulate a large number of common target genes [22, 41]. The in silico data we present here correlate very well with the actual responsiveness of the *SAUR10*-clade to auxin, BR and light. The combination of all three binding motifs (ARF, BZR and PIF) is only present in the promoters of *SAUR10*, *SAUR16* and *SAUR50*, while *SAUR12* lacks an ARF binding site (based on our results and previously published ChIP-seq data; see Additional file 2: Table S1). Despite the induction of *SAUR9* bij BR, we did not identify a BZR binding motif in the *SAUR9* promoter, nor has *SAUR9* be detected as a BZR1 target by Oh et al. [22], suggesting that the BR-induction of *SAUR9* does not occur via BZR1, or that a distant enhancer motif is involved. Comparison of our *SAUR* classification with transcriptome data of the *pifq* mutant [9], supports the link between PIF and ARF regulation, as 12 of the 14 genes upregulated in the *pifq* mutant belong to the Class I *SAURs* and only 2 to the Class II *SAURs*. Our data thus provide more evidence for the interaction between ARF-BZR-PIF, and it will be interesting to investigate further requirements for this interaction, for example a certain spacing between the different cis-elements. The combined AuxRE element and the BZR1 binding site in the *SAUR10* promoter are approximately 100 bp apart (−1538/−1640), but whether these concern really functional binding sites and whether this spacing is important remains to be investigated.

### *SAUR* regulation is highly dynamic

We show here that *SAUR*s are not only expressed in seedlings, but have specific expression patterns throughout the plant and appear to a large extent developmentally regulated. Furthermore, many *SAUR* genes respond dynamically to different hormones. In addition to auxin and BR, *SAUR*s can positively or negatively respond to ethylene [23], GA [7] (Additional file 5: Figure S4), ABA (Fig. 3b; [20]) and cytokinin (Fig. 3c). The dynamic response to environmental and hormonal factors is probably assured by the rapid breakdown of both *SAUR* transcripts and SAUR proteins ([1, 2] and Additional file 5: Figure S4). As soon as the stimulus disappears, SAUR levels will drop and the plant can return to default growth mode. As shown in Table 1, there are binding sites of different TFs involved in plant development in the upstream regions of the *SAUR10*-clade genes. These TFs likely determine the tissue specific expression of the different *SAUR* genes, whereas the amplitude of this expression can probably be controlled by the response to hormone signalling transcription factors.

The dynamic regulation can complicate phenotypic analyses of T-DNA mutants, as it is well possible that a mutant phenotype can only be observed transiently after application of certain stimuli. Our analysis also revealed that SAUR feedback loops may buffer the effect of up- or down-regulation of other *SAURs* (Fig. 1j), probably also hampering the identification of mutant phenotypes. The recent establishment of CRISPR techniques for plants [42] will hopefully facilitate the discovery of mutant phenotypes, because the small coding region can be directly targeted and it will be easier to generate double or triple mutants in one transformation event.

## Conclusions

*SAUR* genes encode growth regulators that are very important for plant growth and development and allow the plant to dynamically respond to its environment. This response has thus far been mainly investigated in seedlings, but we show here that the Arabidopsis *SAUR10-*clade genes *SAUR8*, *SAUR9*, *SAUR10*, *SAUR12*, *SAUR16*, *SAUR50*, *SAUR51* and *SAUR54* are expressed throughout the plant, each displaying specific expression patterns. While overexpression studies revealed that their protein functions are similar, their different responses to hormones and diverse expression patterns indicate that *SAUR* genes enable the plant to fine tune growth in a variety of tissues in response to internal and external cues. In silico cis-regulatory element analyses additionally revealed that all *SAUR*s contain different sets of cis-elements in their upstream sequences. Furthermore, the Arabidopsis *SAURs* could be divided into two classes based on their auxin responsiveness: Class I genes are induced by auxin and often possess AuxRE, BR and PIF binding elements, while Class II genes are not responsive to auxin. The characterization of *SAUR* gene regulation presented in this study largely contributes to the understanding of plant growth dynamics.

## Methods

### Plant materials and growth conditions

The overexpression and reporter lines were generated in the Col-0 background. SALK [43] and JIC SM [44] T-DNA lines were received from NASC, the FLAG T-DNA lines [45] were received from the IJPB in Versailles. All primers used to genotype the T-DNA insertion lines can be found in Additional file 2: Table S3. Plants were grown in a long-day climate chamber (16/8) at 22 °C on rockwool blocks watered with HYPONeX® solution (1.5 g/l). The LED illumination in the growth chamber resulted in the following light conditions: 87.6 μmol m^−2^ s^−1^ photosynthetically active radiation (PAR); R:FR ratio = 30.1). Reduced R:FR conditions were achieved by supplemental FR (730 nm) irradiation, resulting in a PAR of 83.5 μmol m^−2^ s^−1^, and a R:FR ratio of 1.15. Blue light reduction was achieved by filtering the light through a layer of LEE Medium Yellow 010 filter, resulting in a PAR of 70.4 μmol m^−2^ s^−1^, and containing 1.06789E-02 W/(sqm*nm) irradiance in the blue light spectrum (400–500 nm), compared to 3.84107E-01 W/(sqm*nm) under control conditions.

### Generation of transgenic lines

To generate overexpression constructs, the coding sequences of *SAUR8*, *SAUR10* and *SAUR16* were amplified from genomic DNA (see Additional file 2: Table S3 for all primer sequences), recombined into pDONR221 using the BP reaction (Gateway technology, Invitrogen), and subsequently recombined into the binary vector pK2GW7 [46] using the LR reaction. Reporter constructs for the eight *SAUR10*-clade genes were generated by amplification of approx. 3 kb upstream region from genomic DNA. The fragments were recombined into pDONR221 and subsequently into the binary vector pBGWFS7 [46]. All constructs were transformed to Agrobacterium and introduced into Col-0 plants using floral dip.

### Hormone treatments and qPCR analysis

For the hormone treatments, 8–14 day old seedlings were removed from plates (2.2 g/l Murashige and Skoog (MS) medium, 10% sucrose, 0.9% agar) and incubated in liquid 2.2 g/l MS medium with hormones or in mock (control) medium for 4 h. The following hormone concentrations were used: 5 μM IAA (according to [14]), 1 μM Zeatin [19], 100 μM GA3, 100 μM ABA, 5 μM Brassinolide, 5 μM IAA + 5 μM Brassinolide. After incubation, seedlings were frozen in liquid nitrogen and stored at −80 °C prior to RNA isolation. The RNA was extracted using a CTAB/LiCl protocol or with the the InviTrap® Spin Plant RNA Mini kit (Stratec Molecular), DNase treated with Ambion Turbo DNase (AM1907) and reverse transcribed using the iScript cDNA synthesis kit (BioRad). The qPCR reaction was performed with iQ SybrGreen supermix from BioRad on the BioRad iCycler.

### In silico analyses

Motif searches were performed with 3 kb upstream promoter sequences obtained using the Bulk Data Retrieval tool from TAIR, based on TAIR10 (http://www.arabidopsis.org/tools/bulk/sequences/index.jsp). Class I (*SAURs* induced by auxin) and Class II (*SAURs* not regulated by auxin) genes were selected based on Additional file 2: Table S1 (see Additional file 2: Table S2). In addition to Class I and Class II *SAURs*, a background promoter set was used. This consisted of 500 randomly selected Arabidopsis promoter sequences (excluding the *SAUR* promoters).

MEME [47] was applied to perform de novo motif search in the Class I set, Class II set and a set containing all *SAUR* promoter sequences. The fasta-get-markov tool in the MEME package was used to generate a second order Markov model background from the 500 randomly selected promoter sequences. MEME settings included -mod anr and –revcomp. The maximum number of motifs was 20 and motif widths were between 6 and 18. TOMTOM [48] was applied to the resulting MEME motifs using “Complete scoring” (which takes non-matching parts into account), and MEME motifs were compared with the JASPAR Core plant motif database [49], and with the CIS-BP *Arabidopsis thaliana* database [50].

In addition to the de novo motif search, a set of potentially relevant existing motifs were obtained from literature [38, 51] and from the JASPAR database (BZR1, MA0549.1; (http://jaspar.genereg.net). These were converted to MAST format and motif hits were obtained in the sequence datasets using the MAST tool from the MEME package. Searches for consensus patterns were performed using ps_scan [52]. To test for the significance of differences in motif occurrences between different sequence sets, fisher exact test was applied using the fisher.exact function in R [53].

## Additional files

Additional file 1: Figure S1. UPGMA tree of the Arabidopsis SAUR proteins. (PDF 1406 kb)

Additional file 2: Table S1. Overview of published data showing the response of the individual Arabidopsis *SAURs* to auxin and BR. **Table S2.** Classes of *SAUR* genes based on Additional file 2: Table S1. **Table S3.** Primer sequences. (XLSX 38 kb)

Additional file 3: Figure S2. Analysis of the T-DNA insertion lines. (PDF 209 kb)

Additional file 4: Figure S3. Expression patterns of the *pSAUR:GUS* lines. (PDF 869 kb)

Additional file 5: Figure S4. Dynamics of IAA/BR induction (PDF 132 kb)

Additional file 6: Figure S5. Response of *SAUR10*-clade genes to GA application. (PDF 196 kb)

Additional file 7: Figure S6. The location of GUS expression does not change after IAA-BR treatment. (PDF 373 kb)

## Abbreviations

ABA: abscisic acid
ARF: Auxin Response Factor
AuxRE: Auxin Response Element
BR: brassinosteroids
BZR: BRASSINAZOLE-RESISTANT
DST: DownSTream element
GA: gibberellic acid
IAA: Indole-3-acetic acid
MEME: Multiple Em for Motif Elicitation
PAR: photosynthetically active radiation
PIF: Phytochrome Interacting Factor
qRT-PCR: quantitative real-time polymerase chain reaction
SAS: Shade Avoidance Syndrome
SAUR: Small Auxin Upregulated RNA
TF: Transcription Factor
