# Supplementary figures and images for: Divergent regulation of Arabidopsis SAUR genes: a focus on the SAUR10-clade
Source: BMC Plant Biol. 2017 Dec 19;17:245. doi: 10.1186/s12870-017-1210-4 (PMC5735953; doi:10.1186/s12870-017-1210-4)

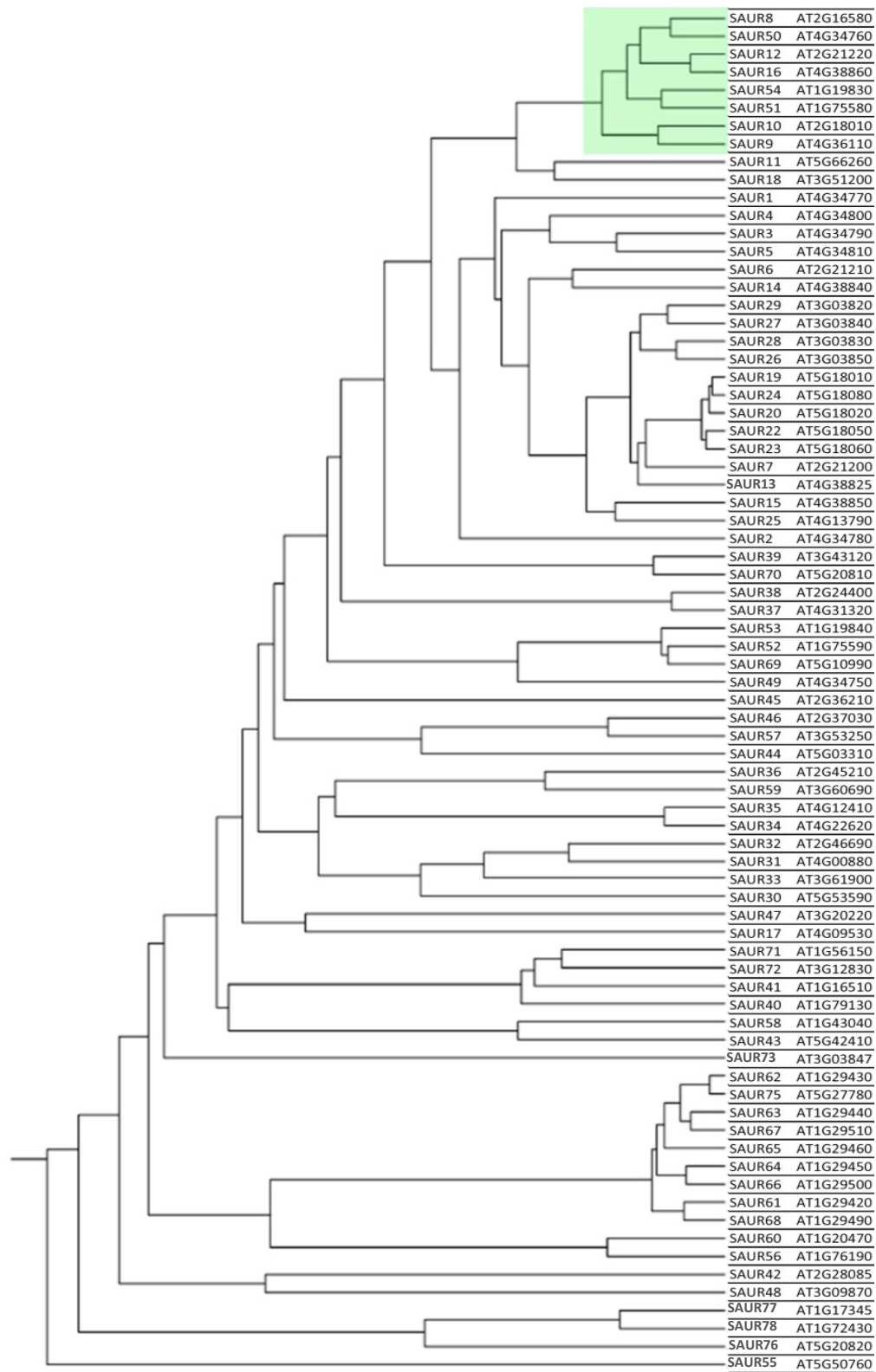

**Additional file 1: Figure S1. UPGMA tree of the Arabidopsis SAUR proteins.**

Supplement: Supplementary file 1 — UPGMA tree of the Arabidopsis SAUR proteins. (PDF 1406 kb) [file 12870_2017_1210_MOESM1_ESM.pdf]
