# Supplementary material for: Divergent regulation of Arabidopsis SAUR genes: a focus on the SAUR10-clade
Source: BMC Plant Biol. 2017 Dec 19;17:245. doi: 10.1186/s12870-017-1210-4 (PMC5735953; doi:10.1186/s12870-017-1210-4)
Supplement: Supplementary file 3 — Analysis of the T-DNA insertion lines. (PDF 209 kb) [file 12870_2017_1210_MOESM3_ESM.pdf]

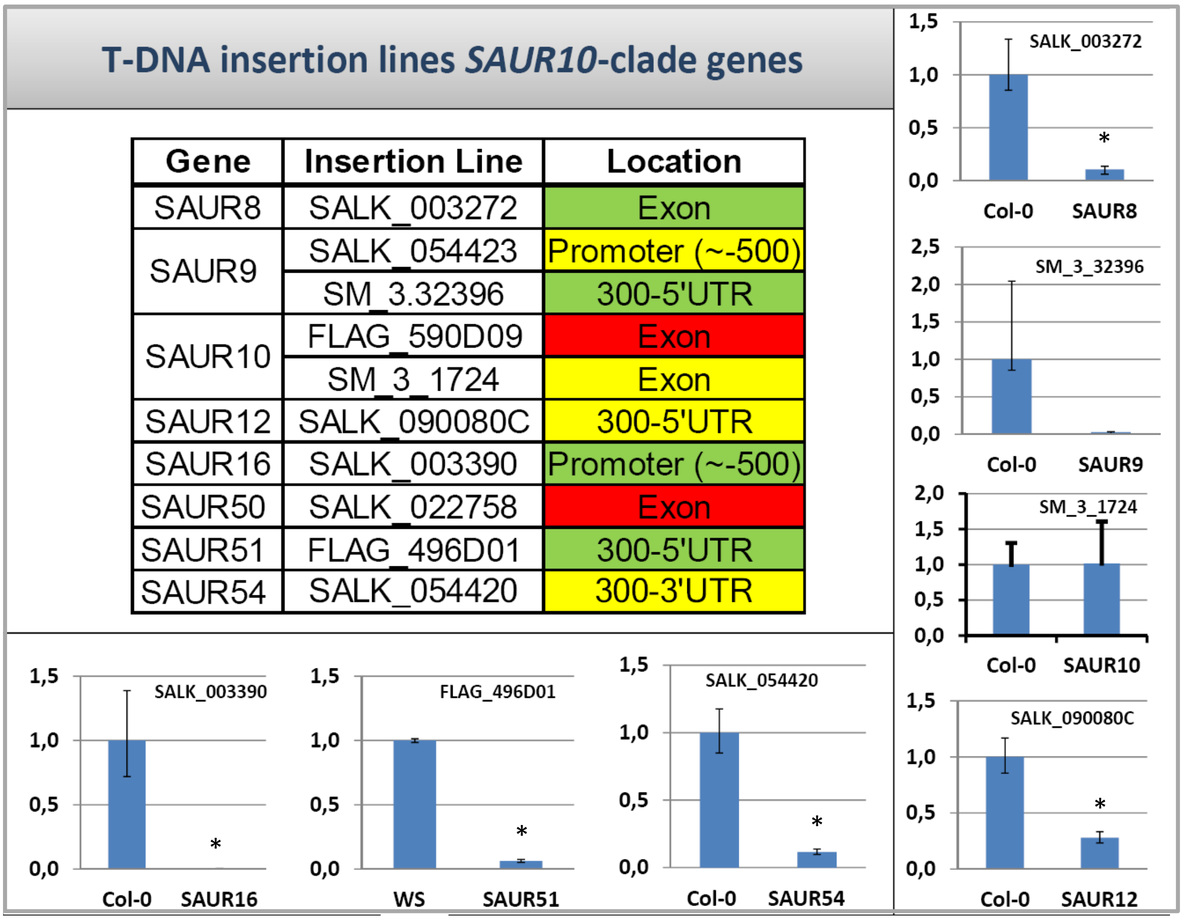

Gene	Insertion Line	Location
SAUR8	SALK_003272	Exon
SAUR9	SALK_054423	Promoter (~-500)
	SM_3.32396	300-5'UTR
SAUR10	FLAG_590D09	Exon
	SM_3_1724	Exon
SAUR12	SALK_090080C	300-5'UTR
SAUR16	SALK_003390	Promoter (~-500)
SAUR50	SALK_022758	Exon
SAUR51	FLAG_496D01	300-5'UTR
SAUR54	SALK_054420	300-3'UTR

Additional file 3: Figure S2. Analysis of the T-DNA insertion lines. The table shows the different lines that were tested. Insertions could not always be identified in the coding sequence, due to the small size of the intronless *SAUR* genes (~350 bp). Green marked lines could be confirmed by genotyping and are probably knock-out lines. Yellow marked lines could be confirmed by genotyping, but are only partially impaired in expression (T-DNA in upstream region). Red marked lines could not be confirmed and do not appear to contain the insertion. The panels on the side show the expression analysis of the *SAUR10*-clade genes in the corresponding insertion lines. The qPCRs were performed with inflorescence tissue. The graph shows the relative fold change compared with the corresponding wild-type line. Significant differences (T-test, p<0.05) are indicated with an asterisk.

The following double and triple mutants were generated: *saur12 saur16; saur50 saur8; saur12 saur16; saur12 saur16 saur54; saur12 saur16 saur50; saur12 saur16 saur8; saur10 saur9*. No aberrant phenotypes were observed in any of these mutants.
