# Supplementary material for: Divergent regulation of Arabidopsis SAUR genes: a focus on the SAUR10-clade
Source: BMC Plant Biol. 2017 Dec 19;17:245. doi: 10.1186/s12870-017-1210-4 (PMC5735953; doi:10.1186/s12870-017-1210-4)
Supplement: Supplementary file 4 — Expression patterns of the pSAUR:GUS lines. (PDF 869 kb) [file 12870_2017_1210_MOESM4_ESM.pdf]

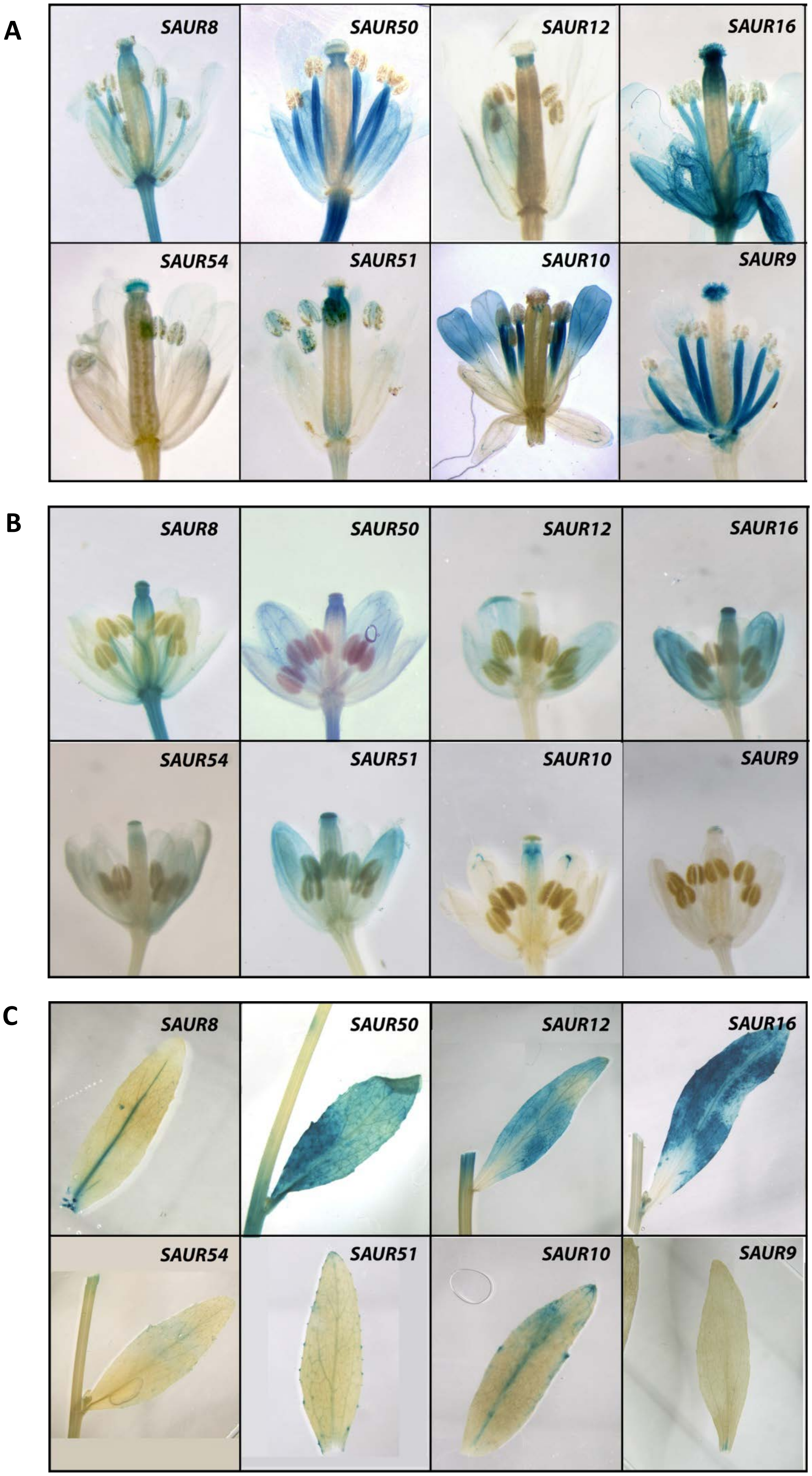
A
SAUR8
SAUR50
SAUR12
SAUR16
SAUR54
SAUR51
SAUR10
SAUR9
B
SAUR8
SAUR50
SAUR12
SAUR16
SAUR54
SAUR51
SAUR10
SAUR9
C
SAUR8
SAUR50
SAUR12
SAUR16
SAUR54
SAUR51
SAUR10
SAUR9

D

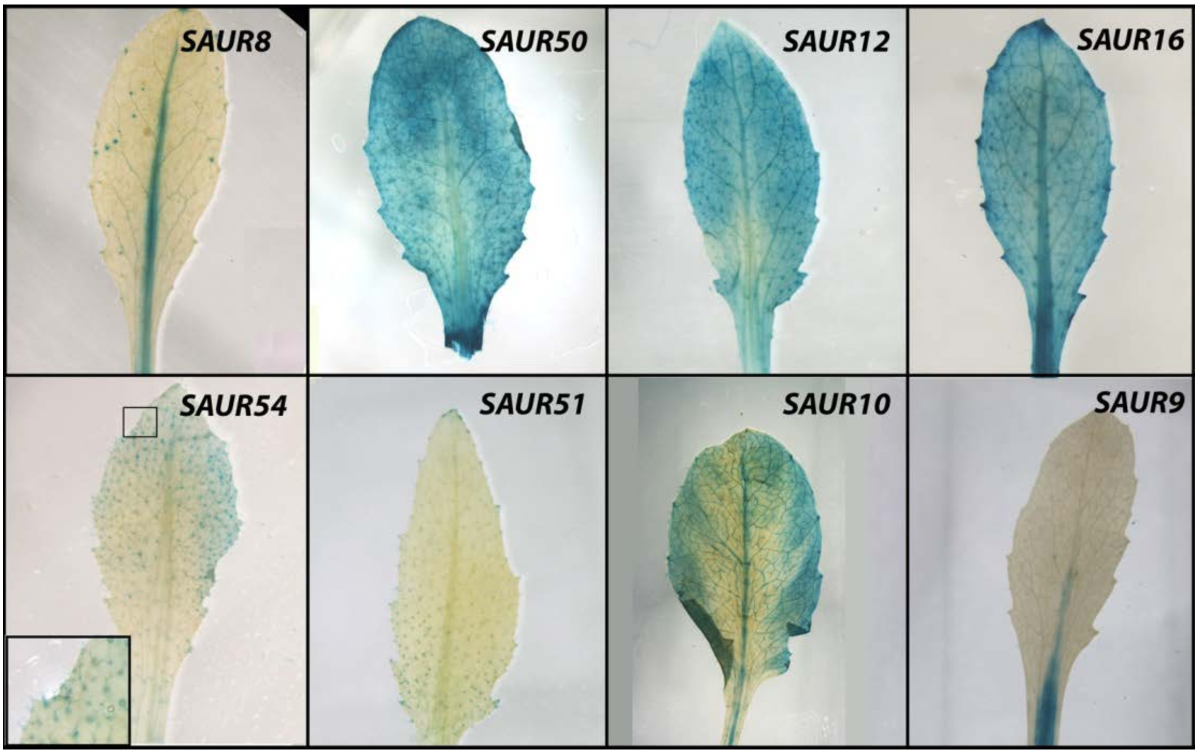
SAUR8
SAUR50
SAUR12
SAUR16
SAUR54
SAUR51
SAUR10
SAUR9

E

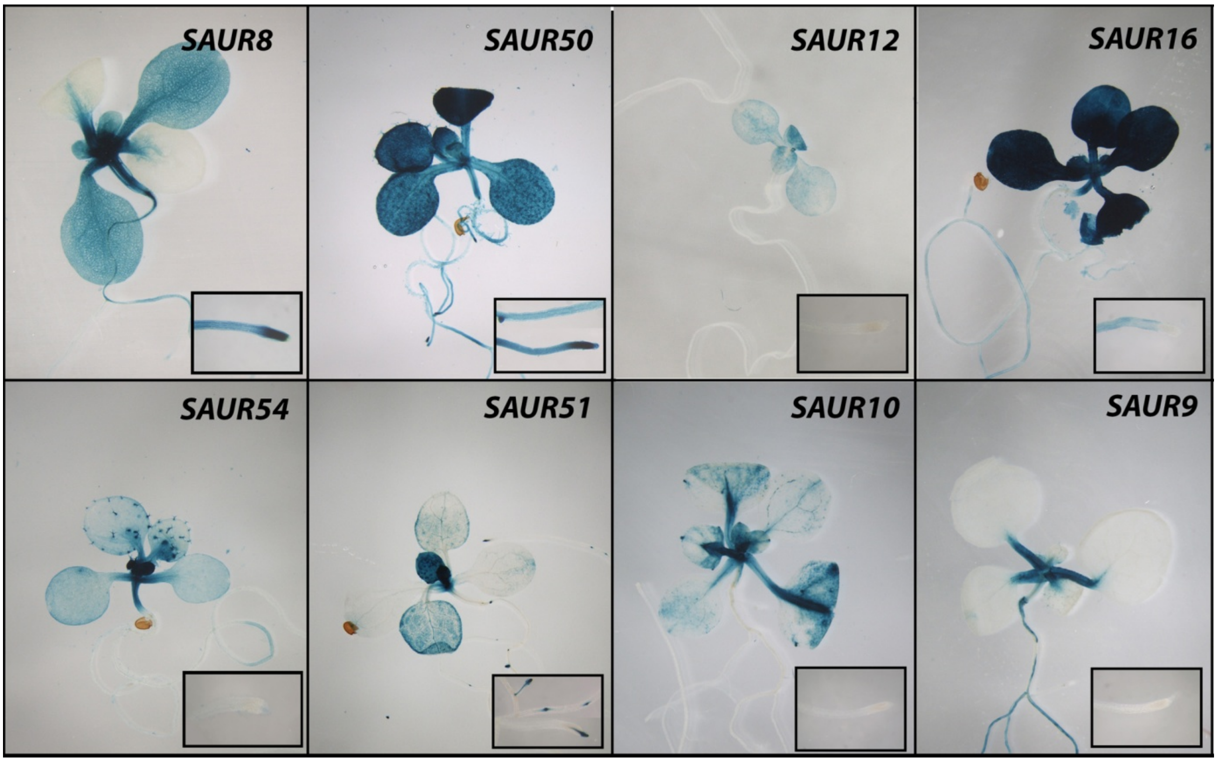
SAUR8
SAUR50
SAUR12
SAUR16
SAUR54
SAUR51
SAUR10
SAUR9

F

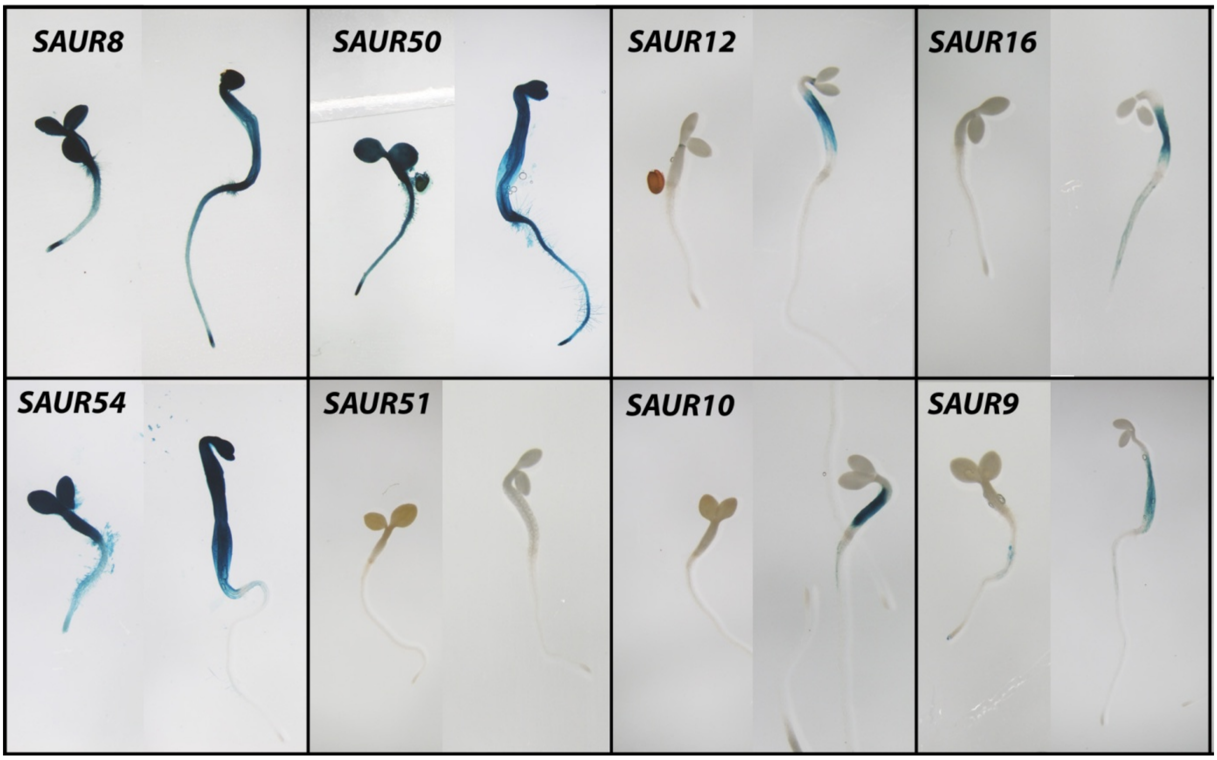
SAUR8
SAUR50
SAUR12
SAUR16
SAUR54
SAUR51
SAUR10
SAUR9

G

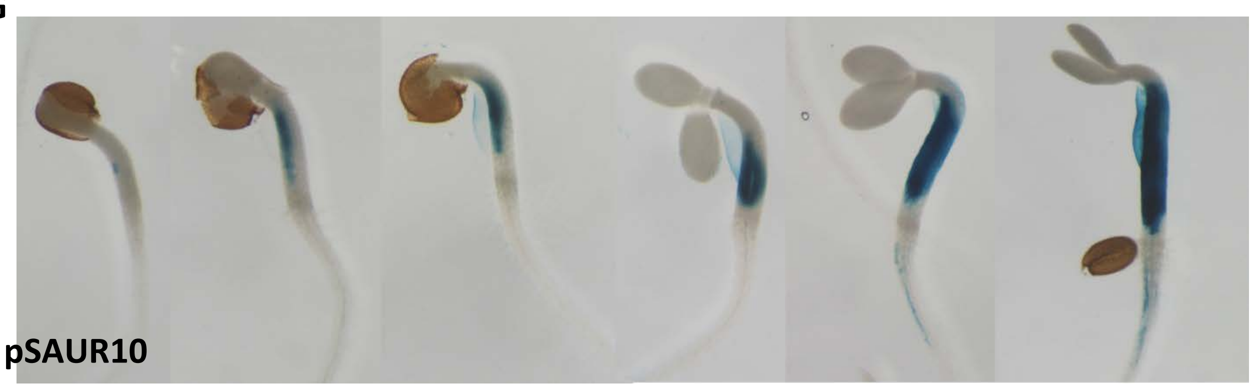
pSAUR10

H

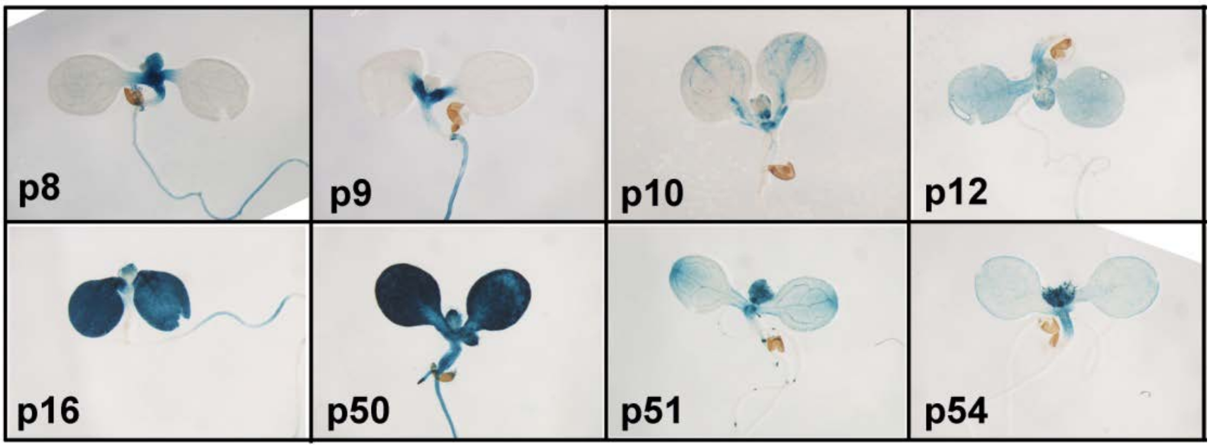
p8
p9
p10
p12
p16
p50
p51
p54

I

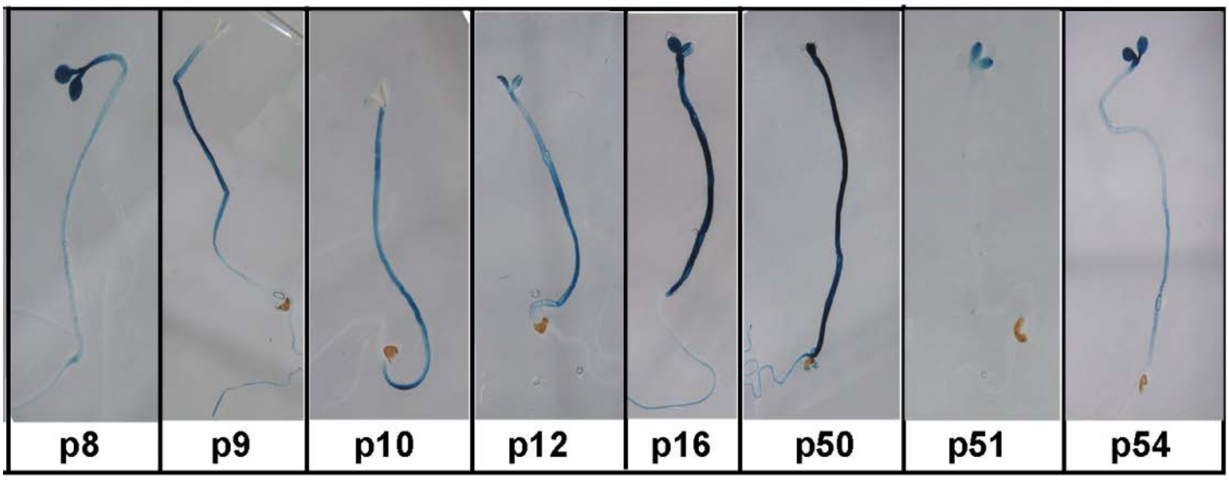

J

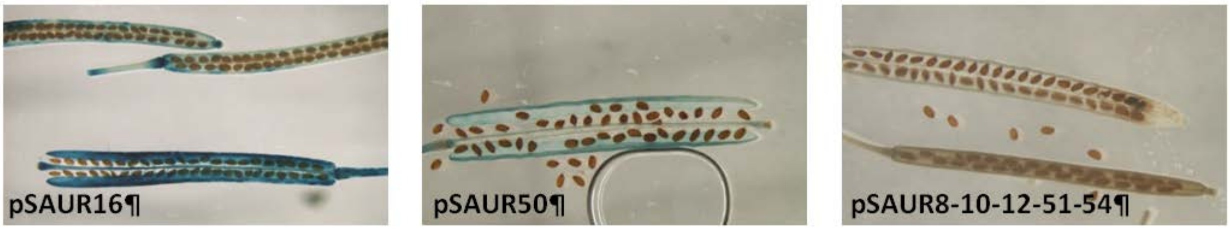

Additional file 4: Figure S3. **Expression patterns of the *pSAUR:GUS*lines**. For every construct, at least two transgenic lines were tested. The tissue was stained in GUS buffer overnight. **A)** Mature flowers (stage 13), **B)** Flowers stage 11, **C)** Cauline leaves, **D)** Rosette leaves, **E)** Seedlings at 12 DAS (days after stratification), **F)** Seedlings 4 DAS. In each panel, the left depicted seedling was light-grown and the right one dark-grown. **G)** *pSAUR10* seedlings 2-4 DAS. For *pSAUR10*, a specific staining pattern in etiolated seedlings was observed, starting with a spot on one side of the hypocotyl and building up to expression throughout the hypocotyl. **H)** Light-grown seedlings at 7 DAS **I)** Etiolated seedlings at 7 DAS, **J)** Siliques stage 18. pSAUR16 and pSAUR50 show staining in this stage, while the other reporters are not active.
