# Supplementary material for: Divergent regulation of Arabidopsis SAUR genes: a focus on the SAUR10-clade
Source: BMC Plant Biol. 2017 Dec 19;17:245. doi: 10.1186/s12870-017-1210-4 (PMC5735953; doi:10.1186/s12870-017-1210-4)
Supplement: Supplementary file 5 — Dynamics of IAA/BR induction (PDF 132 kb) [file 12870_2017_1210_MOESM5_ESM.pdf]

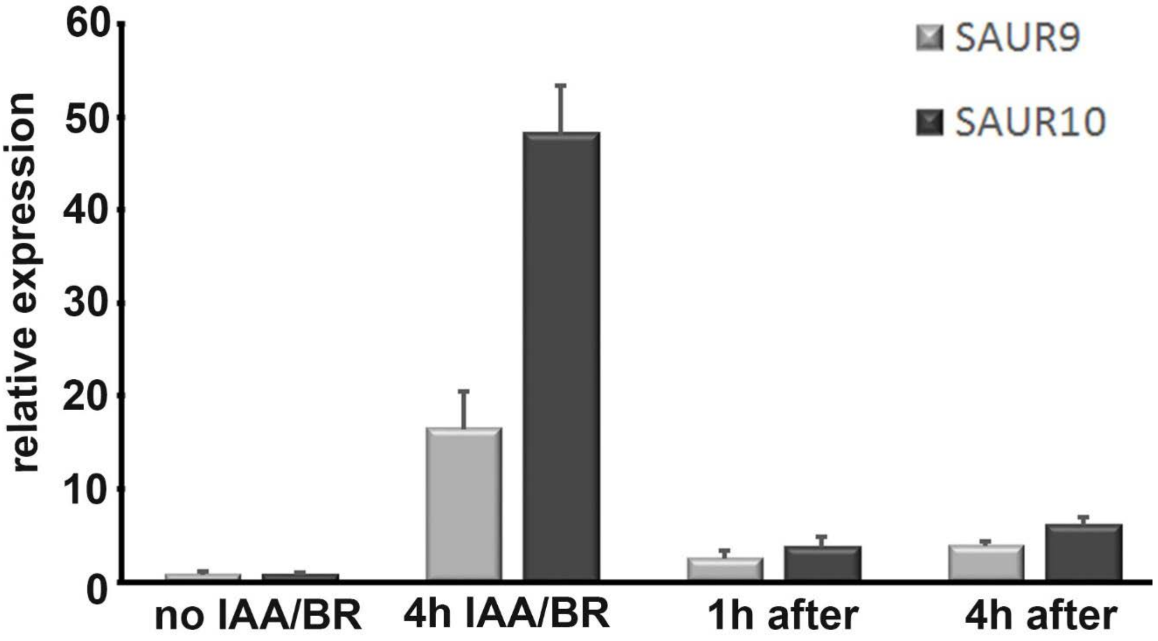

Additional file 5: Figure S4. **Dynamics of IAA/BR induction**. Expression of *SAUR9* and *SAUR10* after 4h of IAA/BR treatment in 10-day old seedlings, and transcript decrease after subsequent replacement of the induction medium by control medium (0.5 MS). No IAA/BR: Seedlings grown for 4 hrs in liquid control medium, 4h IAA/BR: seedlings grown for 4 hrs in IAA/BR medium. 1h after: seedlings grown for 4h in IAA/BR and subsequently for 1h in control medium. 4h after: seedlings grown for 4h in IAA/BR and subsequently for 4h in control medium. The error bars represent the SE based on three biological replicas.
