# Supplementary material for: Divergent regulation of Arabidopsis SAUR genes: a focus on the SAUR10-clade
Source: BMC Plant Biol. 2017 Dec 19;17:245. doi: 10.1186/s12870-017-1210-4 (PMC5735953; doi:10.1186/s12870-017-1210-4)
Supplement: Supplementary file 6 — Response of SAUR10-clade genes to GA application. (PDF 196 kb) [file 12870_2017_1210_MOESM6_ESM.pdf]

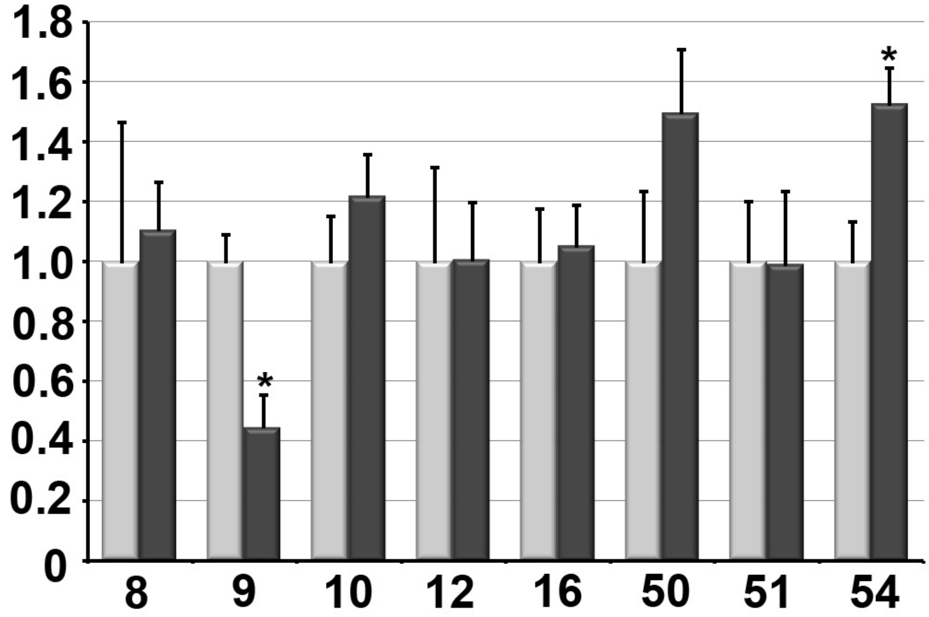

Additional file 6: Figure S5. **Response of *SAUR10*-clade genes to GA application**. Expression of the *SAUR10*-clade genes in seedlings after 4 hours of 100 µM GA3 treatment relative to the expression in mock treated seedlings. The error bars represent the SE based on three or four biological replicas. Significant differences (t-test, $p<0.05$) are indicated with an asterisk.
