# Supplementary material for: Divergent regulation of Arabidopsis SAUR genes: a focus on the SAUR10-clade
Source: BMC Plant Biol. 2017 Dec 19;17:245. doi: 10.1186/s12870-017-1210-4 (PMC5735953; doi:10.1186/s12870-017-1210-4)
Supplement: Supplementary file 7 — The location of GUS expression does not change after IAA-BR treatment. (PDF 373 kb) [file 12870_2017_1210_MOESM7_ESM.pdf]

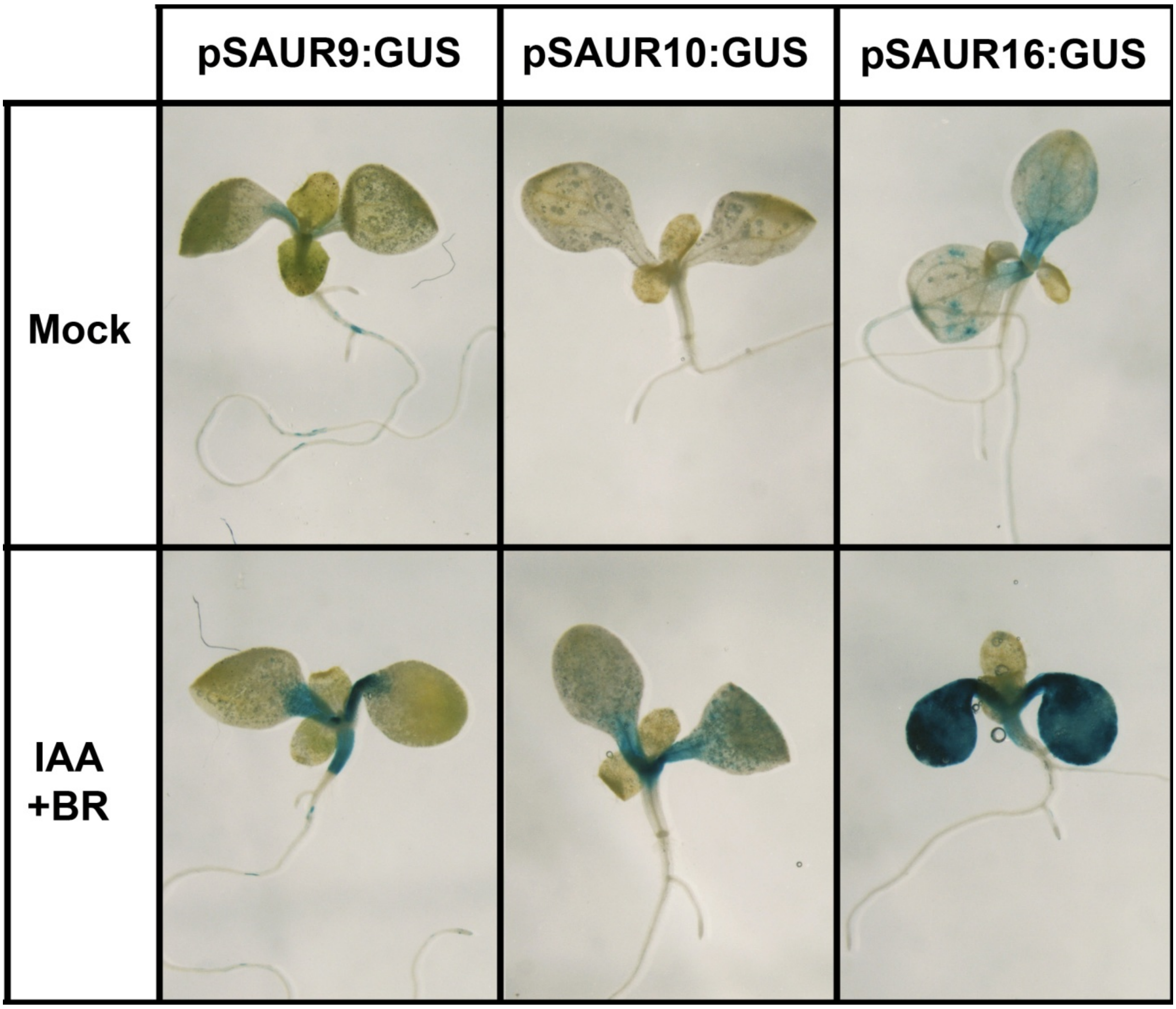

Additional file 7: Figure S6. The location of GUS expression does not change after IAA-BR treatment. Location of the GUS signal in 8-day old seedlings of *pSAUR9:GUS*, *pSAUR10:GUS* and *pSAUR16:GUS* after 4 hrs of IAA-BR treatment compared to 4 hrs of Mock treatment. To visualize the difference in GUS intensity, the seedlings were stained in GUS buffer for 4 hrs only.
